# Investigating the meaning of ‘good’ or ‘very good’ patient evaluations of care in English general practice: a mixed methods study

**DOI:** 10.1136/bmjopen-2016-014718

**Published:** 2017-03-02

**Authors:** Jenni Burt, Jenny Newbould, Gary Abel, Marc N Elliott, Julia Beckwith, Nadia Llanwarne, Natasha Elmore, Antoinette Davey, Chris Gibbons, John Campbell, Martin Roland

**Affiliations:** 1 Cambridge Centre for Health Services Research, Primary Care Unit, Institute of Public Health, Forvie Site, University of Cambridge School of Clinical Medicine, Cambridge, UK; 2 University of Exeter Medical School, Exeter, UK; 3 Distinguished Chair in Statistics; Senior Principal Researcher, RAND Corporation, Santa Monica, California, USA

**Keywords:** PRIMARY CARE, QUALITATIVE RESEARCH

## Abstract

**Objective:**

To examine concordance between responses to patient experience survey items evaluating doctors' interpersonal skills, and subsequent patient interview accounts of their experiences of care.

**Design:**

Mixed methods study integrating data from patient questionnaires completed immediately after a video-recorded face-to-face consultation with a general practitioner (GP) and subsequent interviews with the same patients which included playback of the recording.

**Setting:**

12 general practices in rural, urban and inner city locations in six areas in England.

**Participants:**

50 patients (66% female, aged 19–96 years) consulting face-to-face with 32 participating GPs.

**Main outcome measures:**

Positive responses to interpersonal skills items in a postconsultation questionnaire (‘good’ and ‘very good’) were compared with experiences reported during subsequent video elicitation interview (categorised as positive, negative or neutral by independent clinical raters) when reviewing that aspect of care.

**Results:**

We extracted 230 textual statements from 50 interview transcripts which related to the evaluation of GPs' interpersonal skills. Raters classified 70.9% (n=163) of these statements as positive, 19.6% (n=45) neutral and 9.6% (n=22) negative. Comments made by individual patients during interviews did not always express the same sentiment as their responses to the questionnaire. Where questionnaire responses indicated that interpersonal skills were ‘very good’, 84.6% of interview statements concerning that item were classified as positive. However, where patients rated interpersonal skills as ‘good’, only 41.9% of interview statements were classified as positive, and 18.9% as negative.

**Conclusions:**

Positive responses on patient experience questionnaires can mask important negative experiences which patients describe in subsequent interviews. The interpretation of absolute patient experience scores in feedback and public reporting should be done with caution, and clinicians should not be complacent following receipt of ‘good’ feedback. Relative scores are more easily interpretable when used to compare the performance of providers.

**Strengths and limitations of this study**

- This study is the first to investigate the qualitative meaning of response categories used in patient experience surveys by comparing evaluations of care reported on questionnaires to those reported in interview.
- Questionnaires were completed immediately postconsultation to minimise recall bias, and interviews used video recordings of consultations to prompt patient reflections on care, and were thus not reliant on patients' subsequent recall of events.
- The context of our study limits extrapolation to other settings: our focus was on the items and response options used in the UK General Practice Patient Survey, and the qualitative meanings of labels used in alternative surveys require further exploration.
- The positive skew to our data means we were not able to study the relationship between the choice of negative response options and subsequent interview narratives.
- Our analysis relied on our raters' classification of the sentiment of statements: we used a standard approach to expert consensus rating, but it is possible that different raters would have made different judgements.

## Introduction

Evaluations of patient experience are used worldwide to measure the quality of primary and secondary care.^1^ Public reporting of patient survey data is increasingly the norm, with the expectation that this will foster patient choice and drive improvements in provider performance.^2^ ^3^ As such, large investments are made in funding national patient experience survey programmes, including the General Practice Patient Survey (GPPS) in England, and Consumer Assessment of Healthcare Providers and Systems (CAHPS) in the USA.^4^ ^5^ Data from these surveys inform monitoring and inspection regimes, including the comparison of providers with respect to the quality of their patients' experiences. For example, England's Care Quality Commission undertakes ‘intelligent monitoring’ of the general practitioner's (GP) practices, assessing whether services are ‘safe, effective, caring, responsive and well-led’.^6^ This monitoring relies heavily on GPPS responses, with key indicators including the percentage of respondents who stated that the GP was good or very good at treating them with care and concern, and involving them in decisions about their care.^7^

Despite psychometric validation of national patient experience questionnaires, studies have highlighted challenges in attaching qualitative meaning to the response options used.^8^ ^9^ A choice of ‘very poor’ or ‘poor’ for an item on a patient experience questionnaire would imply that the patient had received an inadequate standard of care and, indeed, recent evidence suggests that, when patients assign such labels to GP's communication skills within a consultation, this is usually confirmed by the assessments of external clinical raters.^10^ However, external clinical raters sometimes identify poor communication where patients have selected ‘good’ or ‘very good’ response options: such observations suggest that interpretation of response labels may not be straightforward.^10^ Indeed, studies investigating the process of questionnaire completion have highlighted that patients may on occasion struggle to accurately represent their experiences of a consultation on standard survey instruments: it may therefore be naïve to interpret response option labels too literally.^11^ ^12^

The aim of this study was to examine the relationship between the rating patients assigned to doctors on a patient experience questionnaire immediately after their consultation, and the same patients' subsequent interview accounts of their experience of care.

## Methods

We conducted an integrative analysis^13^ of questionnaire and video elicitation interview data from patients attending general practice for a face-to-face consultation with a GP.

### Sample and data collection

We invited a purposive sample of general practices in six areas of England (Cornwall, Devon, Bristol, Bedfordshire, Cambridgeshire and North London) to participate in the study. Our sampling strategy for the overall programme of work, of which this study was part, was to explore accounts of consultations with low and high patient ratings for interpersonal skills.^10^ As more than 90% of patients typically rate consultations as *good* or *very good* for interpersonal skills on the national GP patient survey in England, we targeted practices with poorer than average patient experience scores in order to have adequate sample sizes of consultations rated good or less favourably.^14^ Across the six study areas, we therefore purposively selected only practices below the 25th percentile for mean case-mix adjusted doctor–patient communication score in the 2009/10 national GP patient survey. Practices with at least two registered GPs working at least four sessions a week (0.4 full time equivalent) were eligible. Practice recruitment continued alongside data collection until we had conducted and analysed a sufficient range and depth of patient interviews to generate adequate ‘information power’, as defined by Malterud *et al.*^15^

Data collection took place between August 2012 and July 2014. Researchers approached adult patients on their arrival in participating practices and sought written informed consent to video record their consultation. Immediately following the consultation, the patient was asked to complete a short questionnaire. The questionnaire included a set of seven items taken from the national GP Patient Survey which assesses interpersonal aspects of care (table 1: the interpersonal skills domain) and basic socio-demographic questions (gender, age, health status and ethnic background). Patients were also asked if they would consider participating in an interview about their consultation experiences. We aimed to interview at least one patient per participating GP. When more than one patient expressed interest, we used a maximum variation sampling approach to reflect a mix of patient age, gender and interpersonal skills ratings.

**Table 1:** General practitioner–patient interpersonal skills items

Thinking about the consultation which took place today How good was the doctor at each of the following? Please put an × in one box for each row						
	Very good	Good	Neither good nor poor	Poor	Very poor	Doesn't apply *
Giving you enough time	□	□	□	□	□	□
Asking about your symptoms	□	□	□	□	□	□
Listening to you	□	□	□	□	□	□
Explaining tests and treatments	□	□	□	□	□	□
Involving you in decisions about your care	□	□	□	□	□	□
Treating you with care and concern	□	□	□	□	□	□
Taking your problems seriously	□	□	□	□	□	□

*Considered to be uninformative for the purposes of our analysis.

### Video elicitation interviews

We conducted video elicitation interviews up to 4 weeks after the recorded consultation. In these interviews, participants were shown a recording of their consultation with the GP and asked specific questions relating to the consultation and their questionnaire responses (box 1). The video elicitation technique is an established interview method which allows indepth probing of experience during the interview by enabling participants to ‘relive, recall and reflect’ on their recent consultation.^16^ Interviewers paid particular attention to interpersonal aspects of care, asking participants to reflect on how they had chosen their questionnaire response for each of the seven items within the interpersonal skills domain. Thus, for the item ‘How good was the doctor at giving you enough time?’ participants were asked:

- How was ‘*giving enough time’* in this consultation we have just watched?
- Can you tell me what you were thinking about when you answered that question?
- What made you decide this was the right response to give?

Box 1 **The video elicitation interview process**

Data generation focused particularly on participants’ recall of and reflection on the consultation, and how this was expressed in their choice of responses on the questionnaire immediately postconsultation. In each interview, the video of the consultation was used to encourage more accurate recall of specific events during the interaction. Our approach did not aim to establish the facts of what occurred, but rather explored the meaning to patients of actions that were performed in the consultation. We did not seek for patients to rerate their consultation experience again on a questionnaire following their viewing of the consultation video, although narrative re-evaluations did occasionally occur. Instead, we sought to explore the direct experience of each interpersonal aspect of care, using the video as a prompt, and facilitate an explanation of why patients chose that particular questionnaire response option. The interview guide used was semistructured; however, we maintained a tight focus on specific moments and events captured in the recording.

Participants were asked some brief introductory questions about whether they had previously consulted with this doctor, and whether the problem they were consulting about was new or ongoing. Patients were then shown their consultation on the researchers’ laptop. They were encouraged to reflect as they watched the recording. Patients were also given their questionnaire responses and invited to talk through them. The recorded consultation was used as a prompt, enabling further indepth discussion of their experiences in the consultation and their responses to the questionnaire items. Patients were also asked to identify behaviours in the consultation that they considered as contributing to their question responses.

These questions were repeated for all seven interpersonal skills items. In the current paper, we focus only on participants' responses to the first prompt (eg, the statements made following the question, ‘How was ‘giving enough time’ in the consultation we have just watched?’). A separate thematic analysis of the full video elicitation data set will be presented elsewhere. All interviews were audio recorded, with consent and transcribed in full.

### Data analysis: initial coding

Interview transcripts were subjected to content coding. Statements relating to each of the seven interpersonal skills items were identified, extracted and entered into a spreadsheet according to the item they related to. Identified text extracts were subsequently classified independently by two clinical raters (both GPs) as to whether the participant expressed a positive, neutral or negative opinion relating to the doctor's competence and actions in this area. Both raters were blind to the patient questionnaire scores. We used a sentiment analysis approach, which aims to identify the underlying emotional component of language (whether written or spoken), with a focus on classifying positive or negative opinions.^17–19^ It has previously been used, alongside machine learning techniques, to analyse patient feedback online and on social media.^20^ ^21^ Our raters used the following sentiment classifications:^19^

#### Positive

A *positive* text extract exhibited opinions which suggested the doctor's competence or actions in this area were *desirable* or *constructive.*

#### Neutral

A *neutral* text extract exhibited opinions about the doctor's competence or actions in this area *which were not strongly apparent*, with no positive or negative characteristics or features.

#### Negative

A *negative* text extract exhibited opinions which suggested the doctor's competence or actions in this area were of *poor* or *unwelcome* quality.

Raters received the text extracts in a random order: following their independent rating, they met to resolve discrepancies and agreed by consensus a definitive rating of positive, neutral or negative for each extract.

### Data analysis: statistical

Logistic regression models were used to estimate CIs on the prevalence of positive, neutral and negative interview statements overall and according to the score given by the patient. We included only ‘good’ or ‘very good’ scores in these analyses as our aim was to investigate the meaning of positive evaluations only. Because more than one scored text extract came from individual participants, SEs were estimated accounting for clustering by participant. A further logistic regression model investigated the association between non-positive statements and score and item simultaneously (ie, each association was adjusted for the other). Again, SEs accounted for clustering by participant. No account of clustering by GP was made as all observations on participants were nested within GPs.

### Patient involvement

Our programme of research on patient experience, of which this study formed one part, was guided by a lay advisory group. This group, comprising four patient representatives, were involved throughout the conduct of this study, attending regular meetings and providing input and comments via email, post and telephone. They worked with the study research team to identify key questions of concern in the use of patient experience surveys, developing the methods and materials for the patient questionnaire and video elicitation interviews, and reflecting on early findings and analyses and their meaning and implications from patients' perspectives. Dissemination of study findings will take place via lay summaries delivered to participating practices and public reporting on the Cambridge Centre for Health Services Research blog (http://www.cchsr.iph.cam.ac.uk).

## Results

We conducted video elicitation interviews with 50 patients (33 women, 17 men) who had consulted with 32 different GPs in 12 practices. Participants were between 19 and 96 years of age; 20 participants (40%) were aged 65 or above. Ethnic group was reported to be white (n=46), black or black British (n=3) and Asian or Asian British (n=1). In age and gender, interview participants were broadly representative of the primary care patient population responding to patient experience surveys, although minority ethnic groups were under-represented.^22^ Participants were attending for consultations covering a range of conditions, including long-term and acute illnesses. Interviews lasted between 26 and 97 min (median 53 min).

On the postconsultation questionnaires, response options endorsed for GP interpersonal skills items were predominantly positive (table 2). Across the seven items, for the 50 participants, 64% (223 out of 350) were given ratings of ‘very good’. A further 29% were rated as ‘good’ with only 5% rated as ‘neither good nor poor’; no ratings of ‘poor’ or ‘very poor’ were received. Seven ratings were not given (two for ‘explaining tests and treatments’ and five for ‘involving you in decisions about your care’).

**Table 2:** Distribution of response options for interpersonal skills items

	Giving you enough time	Asking about your symptoms	Listening to you	Explaining tests and treatments	Involving you in decisions about your care	Treating you with care and concern	Taking your problems seriously	All items
	N (%)	N (%)	N (%)	N (%)	N (%)	N (%)	N (%)	N (%)
Very good	36 (72.0)	29 (58.0)	38 (76.0)	28 (56.0)	24 (48.0)	35 (70.0)	33 (66.0)	223 (63.7)
Good	13 (26.0)	18 (36.0)	9 (18.0)	17 (34.0)	15 (30.0)	13 (26.0)	16 (32.0)	101 (28.9)
Neither good nor poor	1 (2.0)	3 (6.0)	3 (6.0)	3 (6.0)	6 (12.0)	2 (4.0)	1 (2.0)	19 (5.4)
Poor	0 (0.0)	0 (0.0)	0 (0.0)	0 (0.0)	0 (0.0)	0 (0.0)	0 (0.0)	0 (0.0)
Very poor	0 (0.0)	0 (0.0)	0 (0.0)	0 (0.0)	0 (0.0)	0 (0.0)	0 (0.0)	0 (0.0)
Missing	0 (0.0)	0 (0.0)	0 (0.0)	2 (4.0)	5 (10.0)	0 (0.0)	0 (0.0)	7 (2.0)
Total	50 (100)	50 (100)	50 (100)	50 (100)	50 (100)	50 (100)	50 (100)	350 (100)

We extracted 230 statements from 50 interviews relating to the evaluation of GPs' interpersonal skills. Between one and seven statements were obtained for each participant, with 81% providing five or more statements. The majority of all statements (70.9%, n=163) were assessed to be positive in their evaluations of interpersonal skills; 19.6% (n=45) were neutral and 9.6% (n=22) negative. Seven of the 50 participants gave at least one negative statement during interview. Table 3 shows the classification of statements for each interpersonal skills item.

**Table 3:** Prevalence of positive, neutral and negative comments for different interpersonal skills items

	Giving you enough time	Asking about your symptoms	Listening to you	Explaining tests and treatments	Involving you in decisions about your care	Treating you with care and concern	Taking your problems seriously	All items
	N (%)	N (%)	N (%)	N (%)	N (%)	N (%)	N (%)	N (%)
Positive	32 (82.1)	18 (52.9)	26 (81.3)	14 (51.9)	22 (75.9)	27 (77.1)	24 (70.6)	163 (70.9)
Neutral	4 (10.3)	11 (32.4)	2 (6.3)	10 (37.0)	6 (20.7)	5 (14.3)	7 (20.6)	45 (19.6)
Negative	3 (7.7)	5 (14.7)	4 (12.5)	3 (11.1)	1 (3.4)	3 (8.6)	3 (8.8)	22 (9.6)
Total	39 (100)	34 (100)	32 (100)	27 (100)	29 (100)	35 (100)	34 (100)	230 (100)

The sentiments expressed in interview about that item of care did not always align with the labels of the response options chosen by patients on the questionnaire. Of those who scored their consultation ‘very good’ for an item on the questionnaire, 84.6% (n=132) of statements at interview were classified as positive. However, for those scoring a domain as ‘good’, only 41.9% (n=31) of statements were positive about that aspect of care, and 18.9% (n=14) expressed negative sentiments (table 4). Logistic regression confirmed there was strong evidence of a difference between the prevalence of positive, neutral and negative interview comments and the choice of a ‘good’ or ‘very good’ response on the questionnaire: there was a higher proportion of not-positive (neutral or negative) comments when a ‘good’ response had been chosen (p<0.001) (table 4).

**Table 4:** Prevalence of positive, neutral and negative comments (with 95% CIs) overall and by the box ticked. CIs calculated from logistic regression models which account for clustering of responses by person

		Very good/good combined (n=225)		Very good* (n=151)		Good* (n=74)
	N	% (95% CI)	N	% (95% CI)	N	% (95% CI)
Positive	163	70.9% (60.1% to 79.7%)	132	84.6% (72.7% to 91.9%)	31	41.9% (26.9% to 58.6%)
Neutral	45	19.6% (13.7% to 27.1%)	16	10.3% (5.7% to 17.7%)	29	39.2% (26.0% to 54.1%)
Negative	22	9.6% (4.2% to 20.4%)	8	5.1% (1.7% to 14.7%)	14	18.9% (6.7% to 43.3%)

*Difference between good and very good test from logistic regression p<0.001.

Box 2 presents example text extracts classified as negative when questionnaire responses had been ‘good’ or ‘very good’ for that interpersonal skill. When considering what predicts a ‘not-positive’ statement at interview, logistic regression showed that such statements are much more likely to happen when ‘good’ is ticked on a questionnaire rather than ‘very good’ (p<0.001: table 5). There was also a difference in the probability of interview statements being not-positive according to the item being assessed, with ‘asking about symptoms’ and ‘explaining tests and treatments’ being more likely to predict these (p=0.002) (table 5). We found no evidence that socio-demographic characteristics predicted non-positive statements: we note, however, that we may have had insufficient power to examine socio-demographic variations previously noted in patient responses to questionnaires.^23^ ^24^

**Table 5:** Logistic regression (outcome ‘not-positive’ quote)

		OR (95% CI)	p Value
Box ticked	Very good	Ref	<0.001
	Good	7.63 (2.95 to 19.73)	
Interpersonal skills item	Giving you enough time	Ref	0.002
	Asking about your symptoms	3.82 (1.34 to 10.92)	
	Listening to you	1.30 (0.42 to 4.04)	
	Explaining tests and treatments	4.08 (1.18 to 14.11)	
	Involving you in decisions about your care	1.05 (0.25 to 4.44)	
	Treating you with care and concern	1.43 (0.42 to 4.88)	
	Taking your problems seriously	1.70 (0.59 to 4.89)

Box 2 **Example interview text extracts classified as negative when questionnaire response for relevant interpersonal skills item was ‘very good’ or ‘good’**

“I think he was aware that I had a problem, and he wasn't dismissive of it, and I had the blood test and the X-ray but that's as far as it's gone […] I would have felt that if he'd said ‘Right, make an appointment’ or ‘Come and see your doctor and the information will be passed on’ then I would have felt a little happier about it…but as it is, I'm not.” [27_13_1011]*

“She wasn't very caring and she wasn't concerned about it at all. It was just y'know oh I'll just say this, keep her happy.” **[12_14_4013]**

INTERVIEWER: And involving you in decisions? Do you feel that she did that?

PATIENT: No I don't think she did. No. At all. No I don't think she, no, given me any decisions. She might have directed me into the occupational health side of it but for any other direction I didn't think she helped out. **[12_14_4013]**

“Taking your problems seriously. No, he didn't.” **[53_18_1024]**

“No, he didn't listen to me.” **[53_18_1024]**

“I think he just said it will heal by itself, but I don't know how I'm supposed to know if it's healed or not….He explained, but because I asked questions rather than him just explaining them. […] I don't think he's particularly great…was particularly great at explaining, er, what the problem…em, at what the problem was, it was just, yeah, it's an ear infection, [pause] and that was it.” **[62_11_1010]**

“Well no, he didn't really sort of ask about symptoms.” **[27_13_1004]**

*Patient study ID

## Conclusions

Our results show that when patients rate doctors' interpersonal skills as ‘good’ on a patient experience questionnaire, this does not always indicate positive experiences of care as subsequently described in interviews, and can include important negative experiences. ‘Very good’ response choices are, however, more likely to better reflect almost entirely positive care experiences.

This study was based on a small sample in English general practice, with 50 videotaped consultations. Video elicitation interviews are a labour-intensive approach requiring substantial commitment on behalf of the patient and the researcher, limiting the number that could be undertaken. However, we are confident that our sampling strategy generated sufficient high-quality data to provide a robust assessment of the discordance between questionnaire responses and subsequent accounts of care.^15^ Interviews used the consultation video recording to prompt patient reflections on care, and were thus not reliant on patient recall of events. However, we note that patients may change their views over time: the evaluation of one episode of care may be influenced by subsequent appointments, the course of the illness and by other factors, and so an appraisal of the consultation occurring immediately after the encounter may look different 4 weeks later. A further limitation relates to the context in which this study was conducted: our focus was on the items and response options used in the national GP Patient Survey (such as *very good, good*), and the qualitative meanings of labels used in alternative surveys such as the CAHPS suite of questionnaires (such as *always, usually*) require further exploration.^4^ ^5^ Additionally, the positive skew to our data—in spite of our efforts to purposively seek a range of patient experience scores—means we were not able to study the relationship between the choice of negative response options and subsequent interview narratives. Finally, our analysis relied on our raters' classification of the sentiment of statements: we used a standard approach to expert consensus rating, but it is possible that different raters would have made different judgements.^25^ ^26^ We would add an additional caution about the mixed methods approach we took, and the assumptions inherent in this.^27^ The integrative analysis involved the ‘quantitising’ of qualitative interview data^28^ derived using a method in which patients' recall and reflection raises complex questions about the nature of their assessments of care. The current paper does not address important questions about the nature of the interview data, and—from the perspective of interview content—how participants subsequently account for their choice of response options on feedback questionnaires, whether and how patients consciously reassess questionnaire responses and why accounts of care differ from the choice of response options on questionnaires. These questions are important in furthering our understanding of the benefits and limitations of differing modes of patient feedback; however, they require indepth qualitative data analysis, and cannot be answered using the current integrative approach.

Patient responses to experience surveys are frequently dominated by the most positive response option.^29–32^ However, research using qualitative approaches indicates that experiences may in fact be poorer than those suggested through literal interpretation of the labels of quantitative surveys.^8^ ^9^ Patients may be reluctant to criticise care using survey instruments, a phenomenon that has previously been reported in users of mental health services, and in patients undergoing elective orthopaedic surgery.^11^ ^12^ Previous research comparing quantitative to qualitative accounts of care has centred on analyses of responses to open and closed items within the same questionnaire. For example, in comparisons of free text responses on a US hospital patient experience survey, there was some evidence that negative free text comments may be present even when closed questions were answered positively.^33^ Similarly, analyses of data from a national hospital inpatient survey in Norway, which organised respondents into clusters on the basis of their quantitative responses, found that negative free text comments did occur in association with positive quantitative assessments of care.^34^ Our findings, in which patients were able to reflect on their experiences of care using a questionnaire and during interview, are the first to bring together both of these approaches to gain insights into variations in accounts across these modes.

We suggest two rationales for our current observations. First, our findings may indicate that some patients are inhibited in criticising doctors when providing questionnaire feedback: an inhibition which is weaker in interview settings. Thus, while these patients may recognise lower quality care, they do not report this as such in a questionnaire. Second, in addition to the first rationale, some patients may struggle to evaluate interpersonal care if it is less than very good. Thus, while the most positive experiences may be obvious to patients (reflected in the high concordance between ‘very good’ questionnaire response options and positive interview statements), experiences rated as ‘good’ may be more challenging to evaluate (reflected in the wide range of sentiments expressed at interview, including many neutral statements, in the presence of a ‘good’ questionnaire response option). We note that our analysis identified two questionnaire items (‘asking about symptoms’ and ‘explaining tests and treatments’) which were more likely to predict not-positive interview statements: it is possible that patients find it particularly challenging to evaluate care in these more clinically focussed areas. Regardless of rationale, the rare use of the least positive questionnaire response options seems likely to be reserved by patients to convey notably negative experiences. The literal interpretation of response options on patient experience surveys will present a picture of care which is too positive, with implications for the interpretation of national survey data when used for quality assurance. However, the continued use of such literal interpretations is attractive when it implies—misguidedly perhaps—that the large majority of clinicians being evaluated are delivering high-quality interpersonal care.

These findings suggest that current inspection and monitoring regimes should be reviewed to ensure patient experience surveys are used appropriately in screening for poor care provision. In particular, given that ‘good’ ratings are provided even in the presence of negative experiences, literal interpretations of absolute scores may overstate quality of care if not considered in a fuller context. For example, we might interpret ‘good’ responses as indicating clinicians should not be complacent about the quality of their interpersonal skills: ‘good’ may not necessarily be ‘good enough’. In particular, the practice of combining ‘very good’ and ‘good’ response categories in presenting and summarising feedback may present a misleadingly positive assessment of care quality. In the USA, inpatient patient experience surveys (Hospital Consumer Assessment of Healthcare Providers and Systems (HCAHPS)) are using an alternative approach to address this issue, separating out the most positive ‘always’ response option from that of ‘usually’ in the presentation of questionnaire responses.^35^ Furthermore, given that negative accounts of care are more common when questionnaire responses are ‘good’ rather than ‘very good’, relative approaches to comparing providers, accompanied by defined levels of acceptable performance, are likely to be a more appropriate use of GPPS survey data. We note, for example, that current UK Care Quality Commission ‘intelligent monitoring’ of GP practices uses relative scores in its assessment of variations in performance.^7^ How relative scores can best be used requires further understanding of when questionnaire responses indicate good care is indeed good enough care, and the point at which differences in performance between providers should be of concern. An additional consideration is how best to facilitate quantitative and qualitative evaluations of care alongside new developments in effective analysis of high volumes of textual feedback: systems which encourage both may be able to create a more indepth, nuanced picture of patient experience.^36^ ^37^ In patient-centred healthcare systems, patients should be enabled to reflect candidly on their experiences of care, and be certain that such experiences make a meaningful contribution to quality improvement.
